# Corrosion Performance of ASTM A615 Carbon Steel Bars in Arabian Seawater Under Natural and Simulated Conditions

**DOI:** 10.3390/ma19051035

**Published:** 2026-03-08

**Authors:** Muhammad Wasiq Ali khan, Tehmina Ayub, Sadaqat Ullah Khan

**Affiliations:** 1 Department of Material Engineering, NED University of Engineering & Technology, Karachi 75270, Pakistan; wasiqkhan@neduet.edu.pk; 2 Department of Civil Engineering, NED University of Engineering & Technology, Karachi 75270, Pakistan; tehmina@neduet.edu.pk; 3 Department of Civil Engineering, Thar Institute of Engineering Sciences and Technology, A Constituent College of NED University of Engineering & Technology, Mithi 69230, Pakistan

**Keywords:** ASTM A615 carbon steel, mass loss, diameter loss, corrosion, natural and simulated chloride exposure, predictive model

## Abstract

Reinforcing steel bars in coastal regions are frequently exposed to chloride-rich environments before the concrete placement, yet the mechanical consequences of this pre-embedding exposure are rarely quantified. This study experimentally investigates the corrosion progression and mechanical degradation of ASTM A615 grade 60 reinforcing steel bars subjected to natural marine exposure and accelerated simulated chloride conditions using real Arabian seawater. Bare bars of 10 mm diameter were exposed to outdoor coastal conditions in Karachi and to an electrically accelerated seawater environment. A periodic evaluation was carried out up to 270 days, including visual inspection, mass loss, diameter reduction, tensile testing, and microstructural characterisation using scanning electron microscopy (SEM). Natural exposure produced gradual general corrosion, corresponding to ~0.5% annual cross-sectional loss and minor reductions in tensile strength within experimental variability. In contrast, simulated chloride exposure markedly accelerated deterioration, causing diameter losses approaching 1 mm and reductions in yield and ultimate strength of up to 20–25% within 60 days. Strength degradation trends closely followed section loss, indicating cross-sectional reduction as the dominant observed factor. SEM observations showed porous and cracked corrosion products with limited protective capacity. A performance-based time equivalence between natural and simulated exposure was derived from degradation trends while acknowledging possible mechanistic differences. Regression models relating exposure parameters to residual strength showed strong agreement with experimental data. The findings demonstrate that pre-placement marine exposure can introduce measurable steel degradation, underscoring the need to account for construction-stage corrosion in durability management of reinforced concrete in coastal regions. The findings highlight the critical impact of pre-embedding chloride exposure on reinforcing steel performance and emphasise the need to incorporate construction-stage corrosion effects into durability-based design and marine construction practices.

## 1. Introduction

Construction delays in Pakistan often leave ASTM A615 [1] reinforcing bars exposed to the environment before concrete placement. During this period, corrosion can initiate, particularly in regions influenced by the marine environment. This type of corrosion, which is called “uniform corrosion”, produces a gradual but meaningful degradation. This early exposure commonly results in uniform surface corrosion, where material loss occurs evenly along the bar. As corrosion products occupy more volume than the steel they replace, they generate radial pressure on the concrete, leading to cracking, delamination, and eventual spalling. The combined loss of cross-sectional area and damage to the surrounding concrete reduces strength, ductility, and long-term durability [2]. Basdeki et al. [3] showed that even small reductions in bar diameter can weaken mechanical capacity. Under simulated chloride exposure, Sorbari et al. [4] reported diameter losses of 0.04–0.05 mm and area reductions of 0.5–1.3% after 360 days in 5% NaCl. Other researchers report similar patterns; for example, Almusallam [5] linked 5–15% mass loss to strength reductions of 6–20%, while Apostolopoulos and Papadakis [6] found consistent decreases in yield and tensile strength as corrosion increased. These findings highlight the sensitivity of reinforcing steel to chloride-induced deterioration, even at early exposure stages.

Although uniform corrosion can be estimated when rate data are available, its effects on bond strength and global structural behaviour remain significant. Mitigation measures such as cathodic protection [7], protective coatings [8], and providing extra material [9] become less effective after embedding in the concrete because of the high pH. Huang et al. [7] also observed reduced bond strength when corroded bars were subjected to impressed current protection. Islam [9] also noted that corrosion sensitivity is influenced by microstructure, grain size, and alloying content, along with steel strength grade.

In chloride-rich marine environments, oxygen and temperature levels have a significant impact on corrosion. Oxygen drives the cathodic reaction, but its diffusion is limited in saturated concrete. Splash and tidal zones, however, undergo wetting–drying cycles that enhance oxygen ingress and concentrate chlorides, producing the most aggressive exposure conditions [10]. The temperature accelerates reaction rates and ion diffusion, often doubling the corrosion rate with every 10 °C increase [11]. Corrosion initiates when the chloride levels at the steel surface exceed the threshold needed to disrupt the passive film [12]. A chloride content of approximately 0.4% by cement mass is widely considered critical. Once depassivated, steel experiences a marked rise in corrosion current density as chloride concentration increases [11]. Natural marine splash zones where seawater salts frequently wet the surface often exceed these thresholds, and fully mixed seawater (≈0.5 M Cl^−^) is considered highly aggressive [13]. Across these studies, weight loss, diameter reduction, and mechanical strength deterioration consistently intensify with higher chloride loading and longer exposure. Additionally, after conducting accelerated and simulated corrosion tests on bare bars, Du et al. [14] examined the residual capacity of oxidised bare reinforcing bars while taking into account the effects of reinforcement type and diameter. Their findings demonstrated that the residual cross-section of a corroded bar is no longer round and varies significantly along its perimeter and along its length due to the local chloride attack penetration. The authors of [14] further mentioned that the force–extension curves of corroded and non-corroded bars were alike for up to 16% corrosion, and the residual yield and ultimate forces were decreased more quickly than their average cross-sectional area, with a significant decrease in residual strength.

Other than natural corrosion, there is an impressed current technique for accelerated corrosion testing (ACT) [15,16,17] to complete the investigation within a reasonable amount of time by speeding up natural corrosion in a laboratory through the application of an electrochemical potential between the anode (reinforcing steel) and cathode. This technique is useful for evaluating the effect of exposure to harsh, controlled conditions like salt spray, high humidity, and temperature cycles on protective coatings, materials, and their designs. This approach saves years of outdoor exposure time for industries but is considered effective for comparisons and quality control; however, establishing a perfect correlation of ACT results to real-world service life remains a challenge, leading to the development of more realistic cyclic tests that better mimic actual environmental stresses. A few studies report having used the ACT approach [15,16,17]. For example, El Maaddawy [17] studied the effect of varying impressed current density levels on the actual degree of steel reinforcing bar corrosion using 5% NaCl added to the concrete mix by weight of cement to depassivate reinforcing bars embedded in 150 × 250 × 300 mm reinforced concrete prisms. At the end of the corrosion phase, all corroded reinforcing bars were removed, cleaned following the ASTM G1 [18] standard, and weighed for mass loss estimation, which showed up to 7.27% mass loss. The authors of [17] further stated that the accelerated corrosion process using the impressed current technique was effective in inducing corrosion of the steel reinforcement, and the use of different current densities does not affect the mass loss with respect to Faraday’s law. Sugamoto et al. [16] compared ACT to address concerns related to the corrosion performance of various metal types in a natural atmosphere. Popova and Prošek [15] recently examined the mechanism of carbon steel corrosion in ACT and reported that carbon steel corrosion rates increased during chloride deposition phases due to the formation of poorly protective porous red rust and the continuous supply of fresh chlorides. This finding conflicts with field observations, suggesting that the ACT conditions were overly aggressive and future tests should reduce corrosivity by lowering the temperature and using less concentrated, neutral salt solutions [15].

Despite extensive research on corrosion within concrete, far less attention has been given to the period before embedding. This is important for Pakistan, where ASTM A615 [1] grade 60 bars are commonly exposed to coastal air for extended durations. These bars rely on the alkalinity of concrete to maintain a passive surface film, yet airborne chlorides can depassivate the steel before the construction progresses. Karachi’s humid, saline climate, high chloride deposition rates, and elevated temperatures create severe exposure conditions, further compounded by urban pollution and carbonation. This study addresses this gap by evaluating the corrosion progression and mechanical degradation of ASTM A615 grade 60 steel under both natural coastal exposure and controlled chloride environments. The primary objective of this research is to investigate the effects of pre-embedding uniform corrosion on ASTM A615 grade 60 reinforcing steel bars when exposed to chloride-rich marine environments typical of the Arabian Sea coastline. Unlike conventional studies, which focus on corrosion after concrete embedding, this study emphasises the construction-stage exposure period, which is often overlooked in design and durability assessments.

The specific objectives of this study are to:

1. Quantify the progression of uniform corrosion in reinforcing steel bars subjected to natural coastal exposure and simulated chloride environments using real Arabian Sea water.
2. Evaluate the influence of chloride-induced corrosion on mechanical properties, including yield strength, ultimate tensile strength, and strain capacity, through tensile testing at predefined exposure intervals.
3. Measure physical degradation parameters, such as mass loss and diameter reduction, and establish their relationship with exposure duration and mechanical performance deterioration.
4. Compare natural and accelerated corrosion mechanisms and determine equivalence relationships between exposure duration in simulated and natural environments.
5. Examine corrosion product morphology and microstructural changes using scanning electron microscopy (SEM) to understand rust layer evolution and its protective effectiveness.
6. Provide experimentally validated data to support durability-based design considerations and service-life assessment of reinforced concrete structures constructed in aggressive marine environments.

## 2. Research Significance

This study addresses a critical but insufficiently investigated phase of reinforcement durability: corrosion of steel bars before concrete placement in chloride-rich coastal environments. The majority of current corrosion research focuses on steel embedded in concrete, where the characteristics of the concrete cover and the chemistry of the alkaline pore solution control the corrosion mechanisms. In contrast, the present work examines bare reinforcing bars exposed to natural marine atmosphere and direct seawater conditions during construction-stage storage, a scenario common in coastal regions but rarely quantified in terms of mechanical consequences.

The novelty of this work lies in:

- Establishing a direct experimental link between pre-embedding corrosion damage and residual tensile performance of reinforcing steel.
- Quantifying diameter loss, mass loss, and mechanical degradation under both natural and electrically accelerated chloride exposure using real seawater.
- Demonstrating that pre-placement corrosion produces measurable strength deterioration even before concrete casting, challenging the common design assumption that reinforcement is pristine at embedment.
- Developing predictive regression models that relate exposure duration and corrosion indicators to residual yield and ultimate strength, providing a framework for incorporating construction-stage corrosion effects into durability-based design considerations.

Rather than focusing on corrosion product phase identification, this study emphasises the structural and mechanical implications of early-stage corrosion, offering data relevant to construction practice, storage management, and service-life assessment in marine environments.

## 3. Experimental Programme

In this study, atmospheric corrosion of steel was observed in Karachi, the capital city of Sindh Province in Pakistan. It is located at the geographical coordinates of 24.8607° N, 67.0011° E, having a monsoon climate with hot, rainy summers and cold, dry winters. Based on the Pakistan Meteorological Department [19], the environmental characteristics of Karachi are listed in Table 1, and the average annual concentration of chloride present in the atmosphere is given in Table 2.

### 3.1. Materials

**Figure 1:**
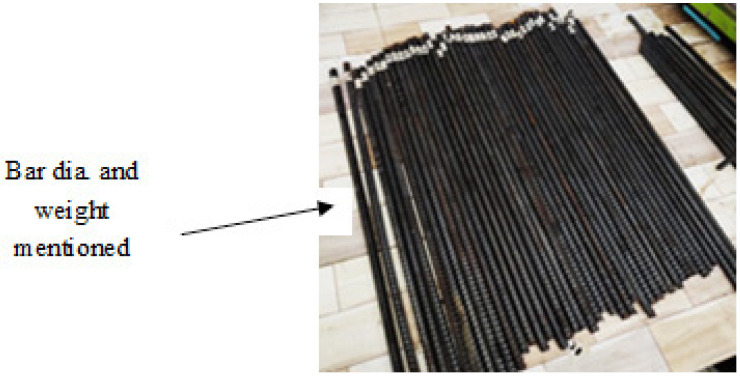
Hot-rolled deformed carbon steel bars of 10 mm diameter that were used.

In this study, a total of 38 hot-rolled deformed carbon steel bars with a diameter of 10 mm and a length of 550 mm were used, which complied with the A615/A615M specifications [1] (refer to Figure 1). Additionally, a power source and actual seawater collected from the coastal region were used in this investigation to provide the bar a constant positive charge to accelerate corrosion.

### 3.2. Exposure Condition

Out of 38 bars (refer to Figure 1), one served as a control (without exposure), whereas 17 bars were exposed to a natural chloride environment, and the remaining 20 were exposed to a simulated chloride environment. Details of both exposure methods are provided in the following section.

#### 3.2.1. Bars Exposed to Natural Chloride Environment

**Figure 2:**
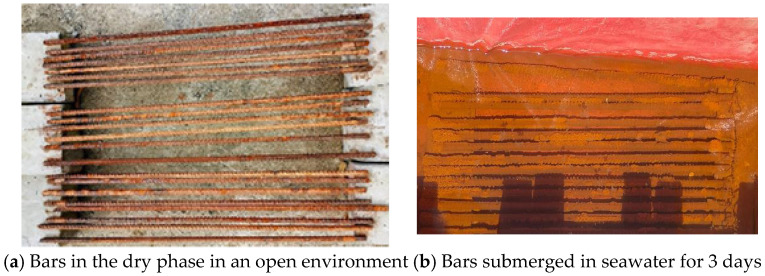
Bars exposed to natural chloride exposure.

A total of 17 bare bars out of 48 were exposed to natural chloride conditions for 9 months. One bar was removed every 15 days from natural exposure for testing. This created a stepped exposure pattern, half a month, one month, one and a half months, and so on. Because the sea level is not constant, the bars were cycled in and out of seawater every 3 days to capture the effects of wetting and drying. Figure 2 illustrates this setup, where Figure 2a shows the bars in the dry phase, and Figure 2b shows them submerged in seawater.

#### 3.2.2. Bars Exposed to Simulated Chloride Environment

**Figure 3:**
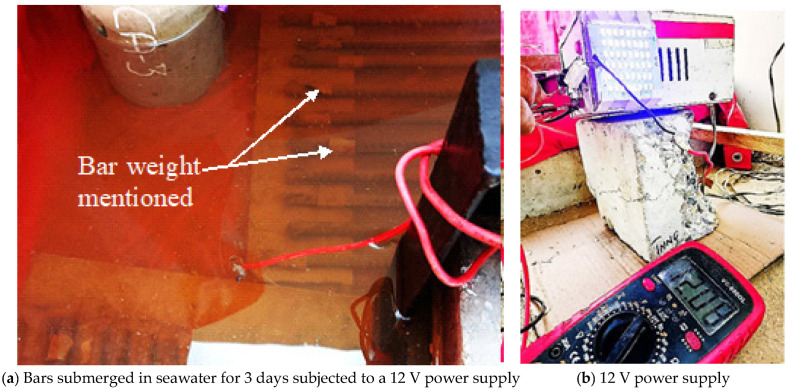
Bars exposed to simulated chloride exposure.

For the simulated chloride exposure, the remaining 20 bars were placed in seawater and connected to a direct power supply set at 12 volt and 2 amper current to accelerate corrosion (refer to Figure 3). The same seawater used in natural exposure was used here to keep the chloride ion concentration consistent. The samples were connected in parallel to the circuit board to maintain a constant voltage of 12 volts throughout the whole experiment [10]. The bars were immersed in a seawater solution to replicate marine conditions. Corrosion was accelerated through the process of electrolysis by applying an external current, thereby inducing a positive charge on the steel surface. The voltage was precisely adjusted and monitored using a digital multi-metre (refer to Figure 3b) to maintain consistent experimental conditions. The bars were completely immersed in seawater, and a current was passed through continuously throughout the exposure period.

### 3.3. Parametric Testing

#### 3.3.1. Chemical Composition of Reinforcement and Seawater

The chemical composition of a 10 mm diameter hot-rolled deformed carbon steel bar was obtained to assess the criteria of meeting the ASTM A615/A615M standard [1]. The chemical composition of seawater used in this study was also determined.

#### 3.3.2. Metallography

**Figure 4:**
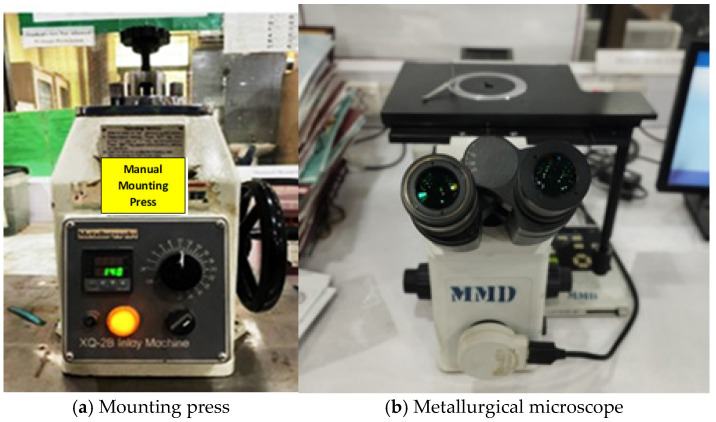
Sample preparation in a mounting press for metallography in a Metallurgical Microscope.

Metallographic samples were prepared in four steps: (1) sample preparation, i.e., mounting, (2) grinding, (3) polishing, and (4) etching; after complete polishing, the face of the sample of steel grade was etched in 2% nital solution. The prepared sample was observed under a metallurgical microscope (refer to Figure 4).

#### 3.3.3. Weight and Diameter Measurement

**Figure 5:**
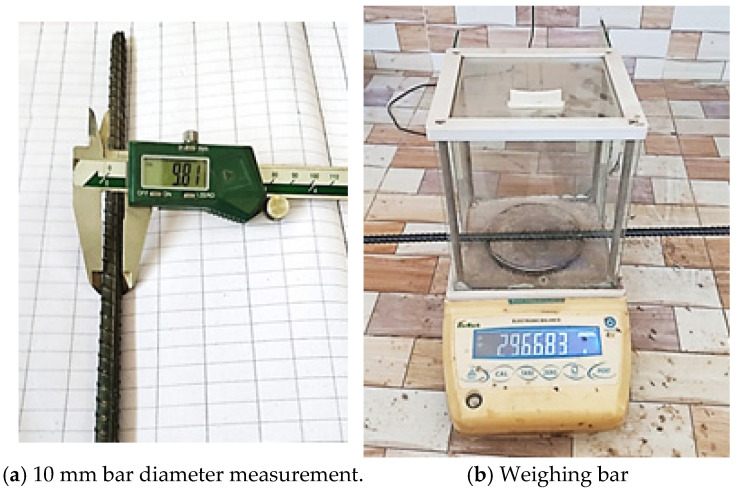
Measurement of bar diameter and weight.

The diameter and weight measurement of a typical bar are shown in Figure 5. A digital Vernier calliper was used, as shown in Figure 5a, to measure each bar’s nominal diameter in mm. These measurements were taken to identify any potential corrosion-related decrease in cross-sectional area after exposure. All bars were also weighed approximately. The weight was measured in grams, as 297 g was divided by the bar length of 550 mm and noted as 0.54 kg/m (refer to Figure 5b). The specimens were then exposed to uniform corrosion in natural and simulated environments.

As mentioned in Section 3.2, one bar was the control bar that had to no-chloride exposure. The control bar and all remaining bars were weighed, and their weight was noted and written on the paper tape with a permanent marker (refer to Figure 1). Similar to pre-exposure measurement (refer to Figure 5), the weight and diameter of the bars were measured and noted after 15 days of natural exposure and 3 days of simulated exposure. This data was used as a benchmark to assess post-exposure corrosion-induced mass loss. Bars were cleaned in compliance with ASTM G1 [18] to eliminate corrosion products following the exposure time. The weights were measured with an accuracy of 1 mg in all measurements. These weights were used to compute the mass loss from corrosion. This process was essential for comparing the weight loss and physical deterioration of bars exposed to corrosive settings to evaluate uniform corrosion. The diameter of the bar after each exposure interval was measured using a digital Vernier calliper. The measurements of weight and diameter were used to quantify the corrosion-related decrease after exposure.

#### 3.3.4. Tensile Testing

**Figure 6:**
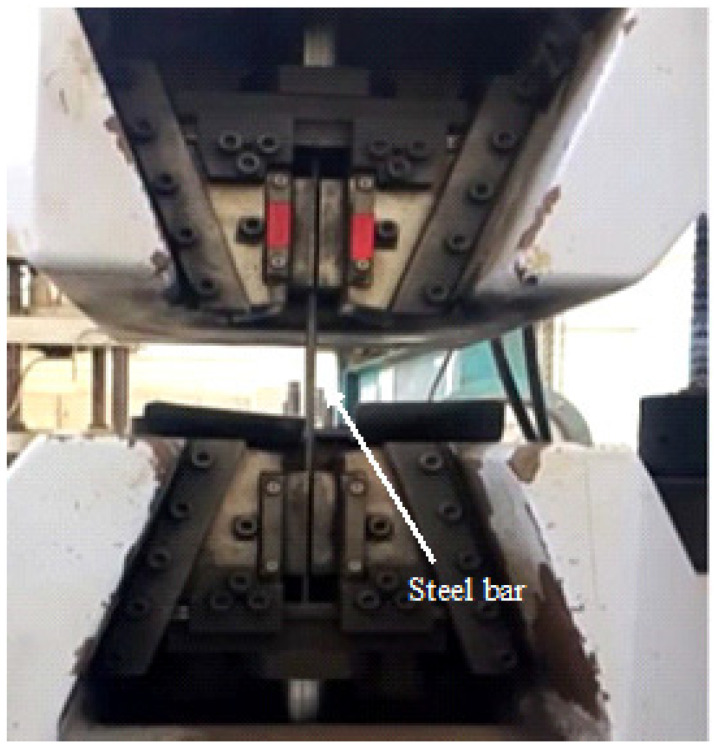
Steel bar before and after exposure, tensile test.

The yield strength, ultimate tensile strength, and elongation of the steel bars were determined using tensile testing. The testing was performed as per ASTM A370 [23] on the steel bars to determine their yield strength, ultimate tensile strength, and elongation prior to exposure, utilising a Universal Testing Machine (UTM) (Kyoto, Japan) with a 1000 kN capacity and a loading rate of 15 mm/min. In addition to establishing a baseline for mechanical characteristics, this was done to examine the impact of uniform corrosion in both natural and simulated environmental settings. The testing setup of steel specimens is shown in Figure 6.

## 4. Results and Discussion

After monitoring and data recording over a 9-month period, the data was analysed in different aspects. The following are the results and their discussion, grouped as unexposed and those under natural and simulated chloride exposure.

### 4.1. Unexposed Condition

#### 4.1.1. Chemical Composition of Reinforcement and Seawater

Table 3 presents the chemical composition of the hot-rolled deformed reinforcing bar. ASTM A615/A615M [1] specifies limits for carbon, manganese, phosphorus, and sulphur, with maximum permissible values defined primarily for phosphorus. The test results indicate that the investigated steel satisfies all ASTM A615/A615M [1] requirements. In particular, the carbon content is below 0.20%, which is within the acceptable range for reinforcing steel and supports adequate ductility.

The chemical composition of Arabian seawater collected near the Karachi coastline is summarised in Table 4. The chloride concentration is significantly higher than values commonly reported for natural seawater [24], indicating a highly aggressive environment. Such elevated chloride levels pose a substantial corrosion risk for reinforcing steel, particularly when bars are exposed prior to concrete placement or directly submerged in seawater.

#### 4.1.2. Metallographic Characteristics

**Figure 7:**
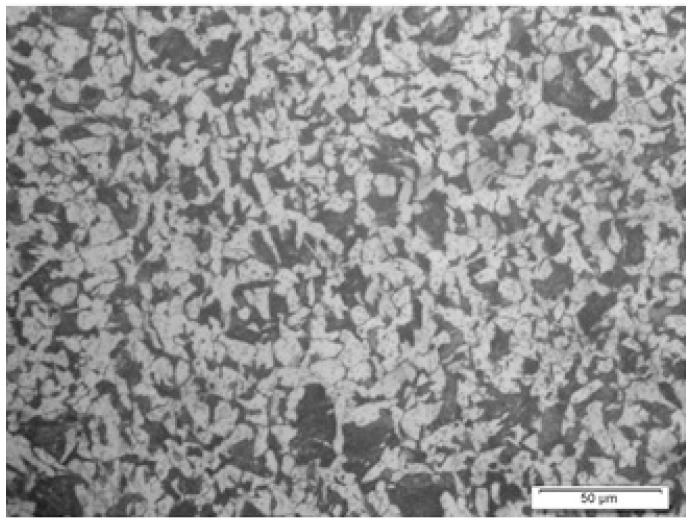
Macrographs showing the section areas of a hot-rolled carbon steel bar.

The optical microstructure of the unexposed reinforcing steel (Figure 7) exhibits a refined and homogeneous ferrite–pearlite morphology typical of low-carbon structural steel. The microstructure consists predominantly of equiaxed ferritic grains with uniformly distributed pearlitic colonies and well-defined grain boundaries. The absence of microcracks, inclusions, or segregation bands indicates sound metallurgical quality and controlled thermomechanical processing. From a corrosion perspective, the ferrite–pearlite heterogeneity plays a critical role in corrosion initiation. Ferrite (α-Fe), being relatively more anodic than cementite (Fe_3_C) present in pearlite, promotes the formation of micro-galvanic cells at ferrite–pearlite interfaces. Under chloride exposure, these local electrochemical potential differences facilitate preferential dissolution of ferritic regions. Grain boundaries may further act as energetically favourable sites for localised attack due to their higher defect density and enhanced diffusivity.

In chloride-laden environments, the passive film formed on the steel surface becomes destabilised once the critical chloride threshold is exceeded. The breakdown of passivity is often initiated at microstructural heterogeneities such as phase boundaries and grain boundaries, where localised chloride accumulation and differential aeration effects intensify anodic reactions. Consequently, pitting corrosion typically nucleates at these susceptible sites before propagating into the ferritic matrix. The observed fine-grain structure may contribute to improved mechanical strength; however, the increased grain boundary area can also increase the number of potential initiation sites for localised corrosion. Therefore, while the unexposed steel exhibits metallurgical uniformity and structural adequacy, its intrinsic ferrite–pearlite heterogeneity governs the early-stage electrochemical response under aggressive chloride conditions.

#### 4.1.3. Tensile Properties of Unexposed Bars

According to ASTM A615 [1], grade 60 reinforcing bars must exhibit a minimum yield strength of 420 MPa and an ultimate tensile strength of 620 MPa. The tested 10 mm diameter bars achieved an average yield strength of 475 MPa and an ultimate tensile strength of 635 MPa, confirming compliance with the standard. These values establish a reliable mechanical baseline for assessing the effects of corrosion under subsequent exposure conditions.

### 4.2. Natural Chloride Exposure

#### 4.2.1. Visual Inspection and Surface Deterioration

Reinforcing bars exposed to the natural marine environment were visually inspected at 15-day intervals for up to 270 days. Corrosion development progressed gradually with increasing exposure duration. After approximately 45 days, a light brown discolouration appeared, indicating the onset of surface oxidation. By 90 days, rust coverage became more pronounced and widespread.

**Figure 8:**
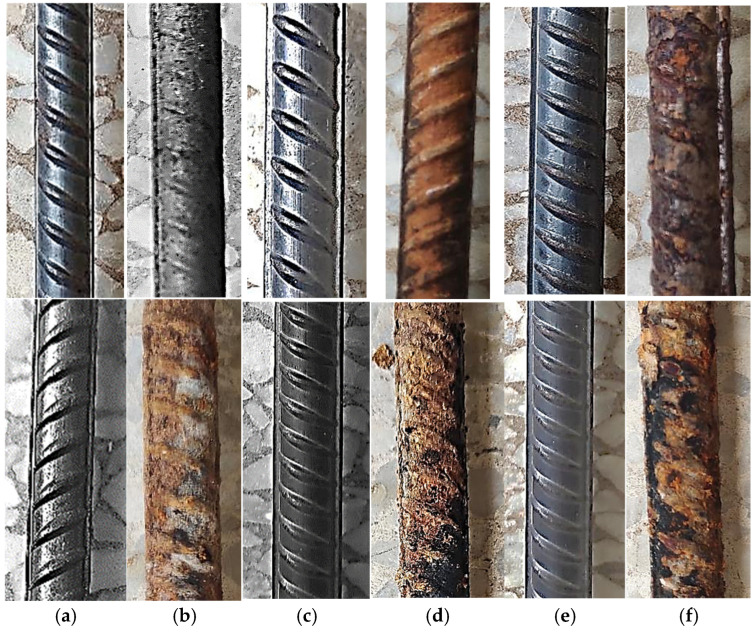
Sequential visual observation of corrosion development on reinforcement bars over 270-days natural exposure period. (**a**) Before and after 45 days of natural exposure; (**b**) before and after 90 days of natural exposure; (**c**) before and after 120 days of natural exposure; (**d**) before and after 150 days of natural exposure; (**e**) before and after 180 days of natural exposure; (**f**) before and after 270 days of natural exposure.

At 120 days, the bars exhibited a continuous reddish-brown rust layer accompanied by increased surface roughness, suggesting ongoing material degradation (Figure 8). Continued exposure beyond 150 days resulted in darker and thicker corrosion layers, while by 270 days, the bars showed extensive rust coverage with flaky and uneven surfaces. These observations confirm a time-dependent progression of uniform corrosion under natural coastal conditions.

#### 4.2.2. Tensile Response After Natural Exposure

Exposure to natural chloride conditions resulted in a gradual reduction in both yield strength and ultimate tensile strength. This reduction is primarily attributed to a decrease in effective cross-sectional area due to corrosion and the development of localised stress concentrations.

**Figure 9:**
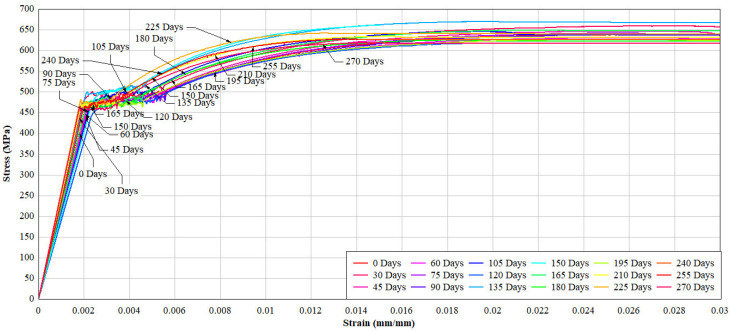
Stress–strain response under tension of reinforcing bars (natural exposure).

**Figure 10:**
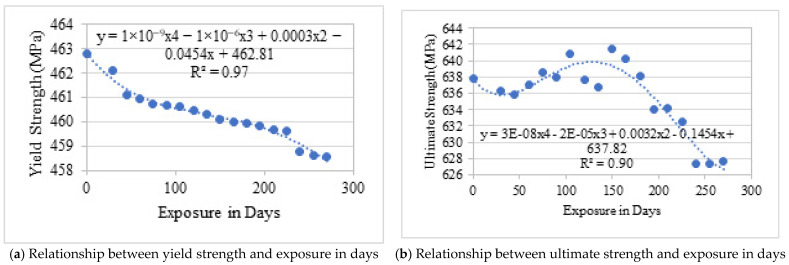
Comparison of yield and ultimate strength with natural exposure.

As summarised in Table 5 and illustrated in Figure 9 and Figure 10, yield strength decreased from 462.8 MPa to approximately 458.6 MPa after 270 days of exposure. The final tensile strength exhibited minor fluctuations throughout the exposure period, which are attributed to experimental variability and possible grip slippage during tensile testing of corroded specimens rather than systematic material behaviour.

Notably, the strain at yielding remained relatively stable, indicating that natural corrosion at this level did not significantly alter elasticity. On average, the reduction in mechanical strength corresponded to approximately 0.5% loss per year, consistent with the measured mass and diameter reductions. The non-linear trend observed in Figure 10 suggests that the formation of a thin rust layer may partially retard corrosion progression during intermediate exposure stages.

#### 4.2.3. Weight Loss Due to Natural Exposure

**Figure 11:**
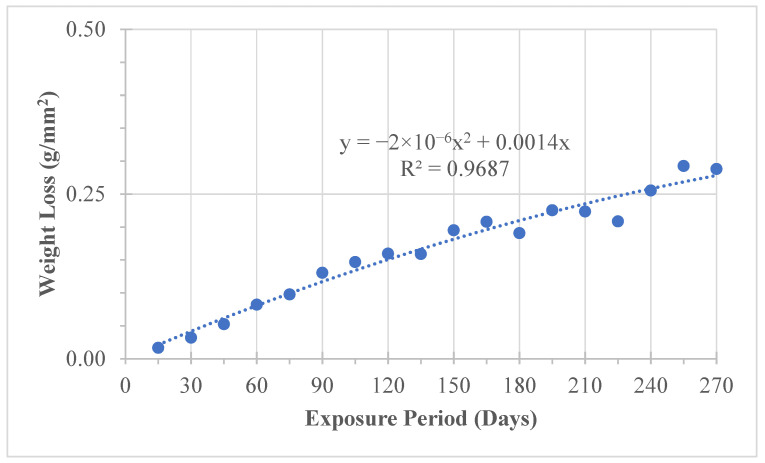
Weight loss versus exposure period under natural chloride exposure.

Material loss due to corrosion was quantified through gravimetric measurements following ASTM G1 [18]. The weight loss data are presented in Table 6 and Figure 11. A progressive increase in mass loss was observed with exposure duration, confirming sustained corrosion activity.

The average corrosion rate under natural exposure was approximately 0.292 g/year, corresponding to an estimated annual reduction of about 0.5% in effective cross-sectional area. This gradual but consistent material loss explains the modest decline in mechanical properties observed in tensile testing.

#### 4.2.4. Diameter Reduction

**Figure 12:**
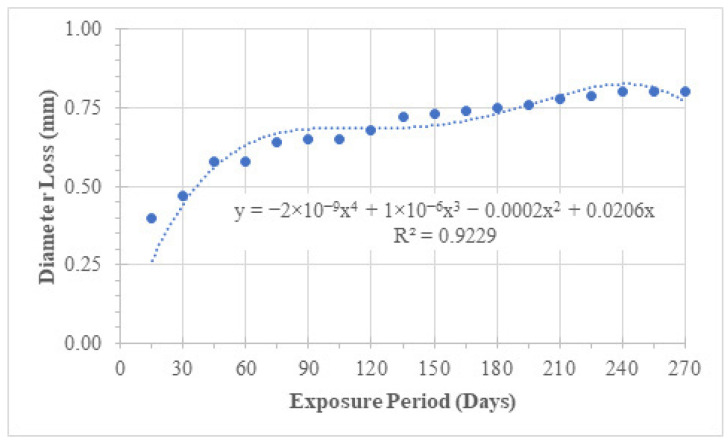
Graphical representation and trending of diameter loss (natural exposure).

Diameter measurements revealed a systematic decrease with increasing exposure time (Table 7 and Figure 12). After 270 days, the average diameter reduction reached approximately 0.8 mm. Although the corrosion was predominantly uniform, small variations along the bar length indicate localised chloride attack.

The measured diameter loss closely correlates with mass loss trends, confirming that section reduction is the dominant mechanism governing strength degradation under natural exposure conditions.

### 4.3. Simulated Chloride Exposure

The primary electrochemical reactions involved during the accelerated corrosion process are as follows:

At the anode (oxidation of iron):

Fe → Fe^2+^ + 2e^−^ (1)

Fe^2+^ → Fe^3+^ + e^−^ (2)

At the cathode (reduction of oxygen in the presence of water):

O_2_ + 2H_2_O + 4e^−^ → 4OH^−^ (3)

The overall result is the formation of iron oxides, commonly referred to as rust, which leads to the deterioration of the steel reinforcement.

#### 4.3.1. Visual Inspection Under Accelerated Conditions

Bars subjected to simulated chloride exposure exhibited rapid corrosion initiation due to the applied impressed current. No visible rust was observed during the first few hours; however, localised corrosion spots appeared along the ribs after approximately 11 h. With increasing exposure time, rust coverage expanded rapidly, forming a continuous reddish-brown layer accompanied by significant surface roughness.

**Figure 13:**
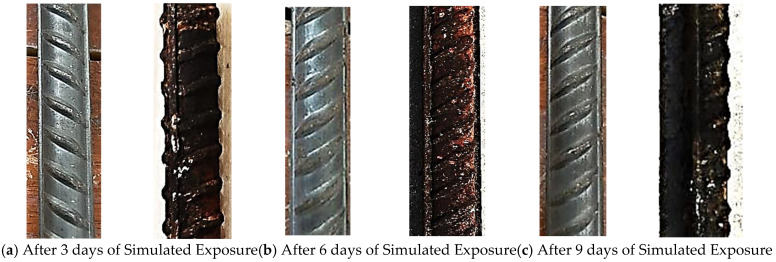
Bars before and after exposure to chloride environment (simulated).

By 30 days of simulated exposure, corrosion severity exceeded that observed after several months of natural exposure, confirming the aggressive nature of the accelerated testing environment (Figure 13).

#### 4.3.2. Tensile Response After Simulated Exposure

**Figure 14:**
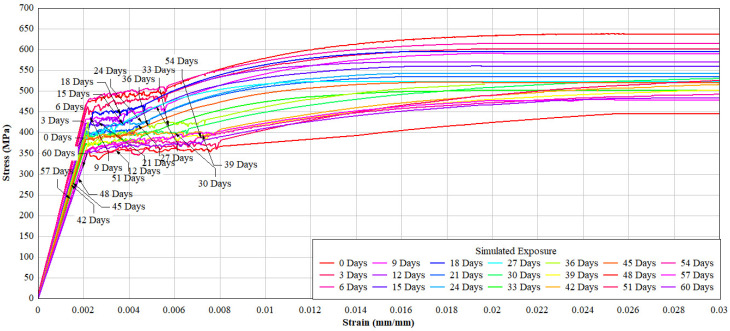
Stress–strain response under tension of reinforcing.

**Figure 15:**
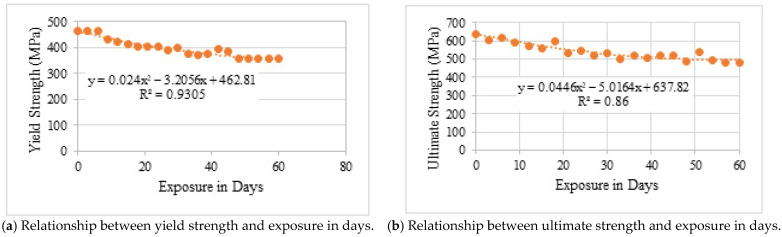
Comparison of yield and ultimate strength with simulated exposure.

Mechanical degradation under simulated exposure was significantly more pronounced than under natural conditions. As shown in Table 8 and Figure 14 and Figure 15, both yield and ultimate tensile strengths decreased sharply from the early stages of exposure.

After 60 days, yield strength decreased by approximately 23%, while ultimate tensile strength declined by up to 25%. These reductions are attributed to rapid cross-sectional loss and severe anodic dissolution caused by the impressed current. On average, strength degradation in one day of simulated exposure was equivalent to approximately 12–25 days of natural exposure, depending on the parameter considered.

#### 4.3.3. Weight Loss Under Simulated Exposure

**Figure 16:**
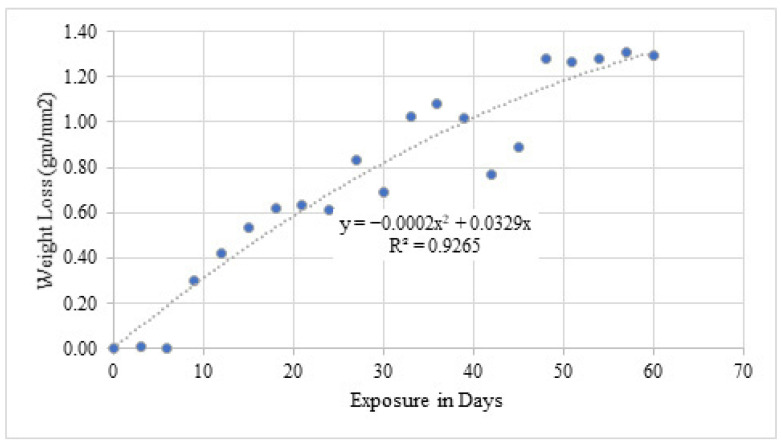
Graphical representation of weight loss due to simulated chloride exposure.

The weight loss results for simulated exposure are presented in Table 9 and Figure 16. A substantially higher corrosion rate of approximately 20.8 mg/day was observed, corresponding to an estimated annual cross-sectional reduction of 12.6%. Compared with natural exposure, the accelerated environment intensified corrosion by more than an order of magnitude.

#### 4.3.4. Diameter Loss Under Simulated Exposure

**Figure 17:**
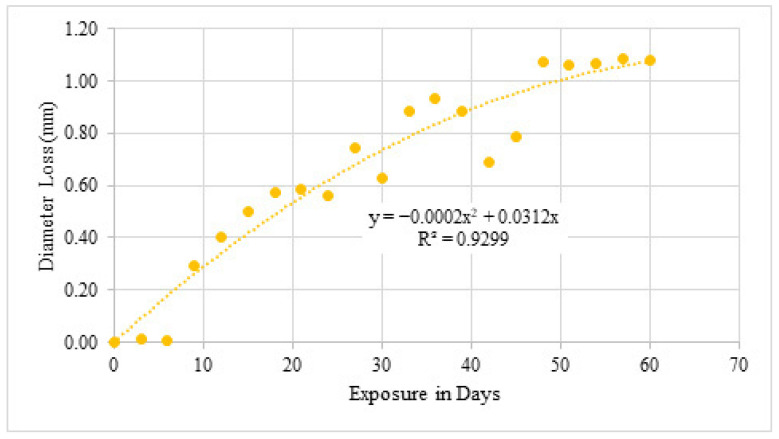
Graphical representation of diameter loss due to simulated chloride exposure.

Diameter measurements (Table 10 and Figure 17) show rapid and substantial section loss. After 60 days of simulated exposure, diameter reduction exceeded 1 mm in several specimens. This aggressive reduction confirms that impressed current exposure effectively accelerates corrosion damage and produces severe section loss within a short period.

## 5. Microscopic Analysis (SEM Results)

**Figure 18:**
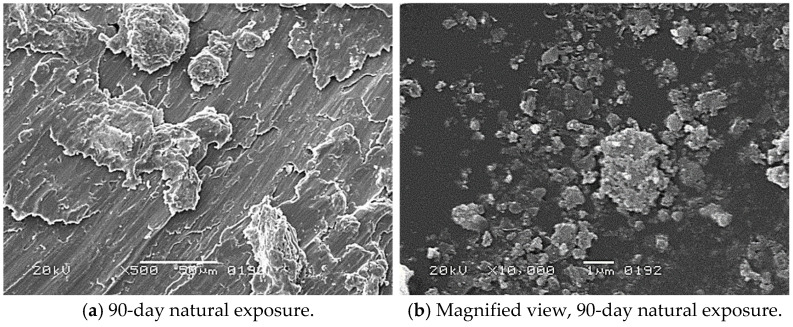

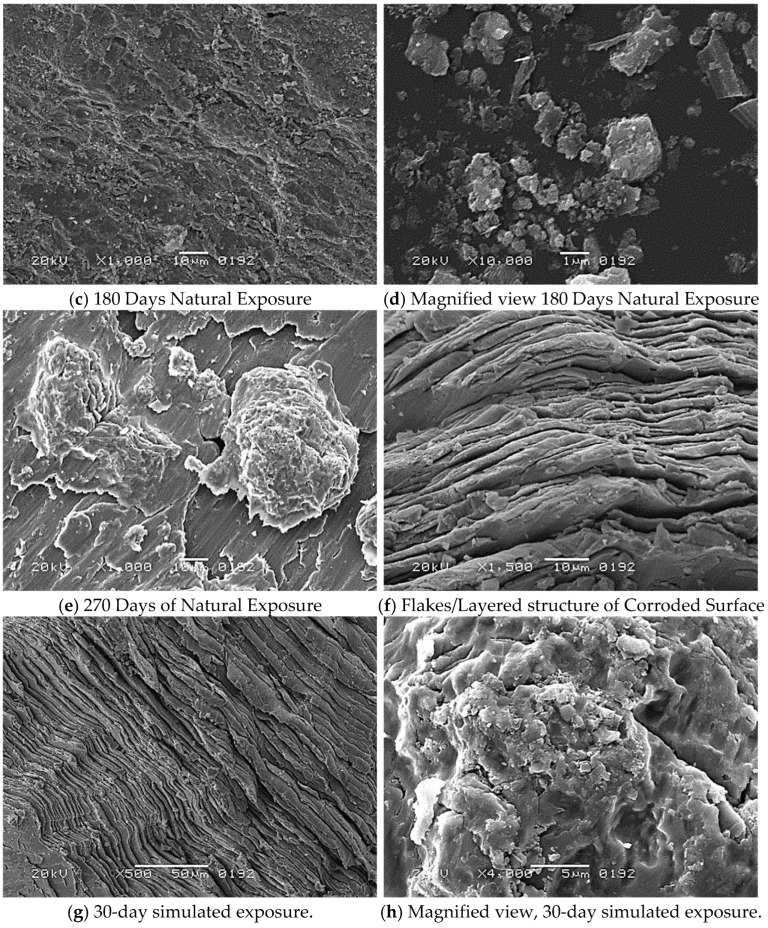
SEM image of bars after natural and simulated chloride exposure.

The corrosion morphology of reinforcing steel bars exposed to natural and simulated chloride environments was examined using scanning electron microscopy (SEM) (Arizona, USA) coupled with energy-dispersive X-ray spectroscopy (EDX).(Arizona, USA) Representative SEM images corresponding to different exposure durations and environments are shown in Figure 18.

### 5.1. Natural Chloride Exposure

After 90 days of natural exposure (Figure 18a,b), the steel surface exhibits early-stage corrosion characterised by localised, discontinuous corrosion products adherent to the substrate. These products appear as irregular nodular agglomerates distributed non-uniformly across the surface, while portions of the underlying steel matrix remain visible. This morphology indicates initial depassivation of the steel surface due to chloride ingress, with corrosion primarily occurring in the form of localised attack.

At 180 days of exposure (Figure 18c,d), the corrosion layer becomes more continuous and compact, covering a larger fraction of the steel surface. Surface roughness increases noticeably, and the original steel texture becomes less distinct. The corrosion products at this stage suggest sustained electrochemical activity, with localised corrosion sites growing and progressively merging. The transition from discrete corrosion spots to a more uniform corrosion layer reflects the combined effect of repeated wetting–drying cycles and continuous chloride availability in the marine environment.

After 270 days (Figure 18e,f), severe surface degradation is observed. Thick, uneven corrosion layers with porous and cracked features dominate the surface. The corrosion products exhibit a flaky, layered morphology, indicating repeated cycles of oxide formation, cracking, and partial spalling. Such rust layers are mechanically unstable and poorly protective, allowing continued access of oxygen and chlorides to the steel surface. This advanced corrosion state is consistent with the measured mass loss, diameter reduction, and gradual strength degradation observed under natural exposure.

### 5.2. Simulated Chloride Exposure

Bars subjected to simulated chloride exposure under impressed current exhibited markedly different corrosion morphology. After only 30 days of exposure (Figure 18g,h), the steel surface shows extensive material dissolution and severe corrosion product formation. Compared to natural exposure, corrosion damage at this stage is substantially more pronounced despite the shorter duration.

The corrosion products formed under simulated conditions appear loose, fragmented, and poorly adherent, with evidence of surface detachment and void formation. This morphology reflects rapid anodic dissolution driven by the applied electrical potential, which suppresses passive film formation and accelerates metal loss. Gas evolution and high current density further contribute to the detachment of corrosion products, continuously exposing fresh steel surface to the aggressive environment.

The observed rust morphology under simulated exposure represents a shift from diffusion-controlled corrosion, typical of natural marine environments, to electrochemically driven corrosion dominated by impressed current effects. This mechanistic difference explains the significantly higher corrosion rates, greater section loss, and pronounced reductions in mechanical strength measured in the simulated exposure tests.

### 5.3. Implications of SEM Observations

Across both exposure conditions, SEM observations confirm that corrosion products formed on bare reinforcing steel do not provide an effective protective barrier. The porous, cracked, and loosely bonded nature of the rust layers facilitates continued ingress of chlorides and oxygen, promoting sustained corrosion progression.

The more severe and unstable corrosion morphology observed under simulated exposure correlates well with the rapid loss of cross-sectional area and the pronounced degradation in yield and ultimate tensile strength. In contrast, the comparatively slower evolution of corrosion products under natural exposure explains the gradual and limited mechanical deterioration observed over the 9-month exposure period.

## 6. Predictive Models for Yield and Ultimate Strength of Bare Bar by Regression

The following expressions have been proposed based on the correlation of variables with the corrosion of bare steel bars:

(4) $$Y=Y_{o}-9\times 10^{-6}Q+2.92\times 10^{-4}C_{cl}-0.573R_{c}T_{exp}$$

(5) $$U=U_{o}-1.2\times 10^{-5}Q-1.95\times 10^{-3}C_{cl}-0.024R_{c}T_{exp}$$

where

Y=yield strength of steel bar after Chloride Exposure (MPa)

$$Y_{o}=yield\;strength\;steel\;bar\;before\;exposure\;(MPa)$$

$$Q=charge\;passed\;in\;steel\;bar,\;which\;is\;Q=IT_{exp}\times 86,400$$

$$T_{exp}=exposure\;period\;in\;days$$

$$C_{cl}=concentration\;of\;chloride\;\left(\frac{mg}{L}\right)$$

$$R_{c}=rate\;of\;corrosion\;in\;MPa/Day$$

U=ultimate strength of steel bar after Chloride Exposure (MPa)

$$U_{o}=ultimate\;strength\;steel\;bar\;before\;exposure\;(MPa)$$

Three major factors contribute to the corrosion of bare steel bars. The first factor is the charge passed, ***Q***, from the bare steel bar under chloride exposure. The term Q can be determined by taking the product of the current in amperes with the exposure period, $T_{exp}$. In this study, the induced current in simulated chloride exposure was 2 amperes, while the current in normal exposure to chloride was observed to be  2 μA. The second term is the chloride concentration, which may affect the corrosion of the bare steel bar. The chloride concentration, $C_{cl}$, as mentioned in Table 2 and Table 4, are 500 mg/L and 20,000 mg/L for normal and simulated exposure. The third one is the corrosion rate, $R_{c}$, which can be determined by integrating the trend line equation in Figure 10 and Figure 15. The corrosion determined was taken as the product of the exposure period, $T_{exp},\;$ and denoted as $R_{c}T_{exp}.$

Predictive models for two prominent stresses of bare steel bar were proposed for the first time, and thus variables are selected based on the correlation to verify the effect of these variables on the corresponding dependent variable. The results of the correlations are mentioned in Table 11.

**Figure 19:**
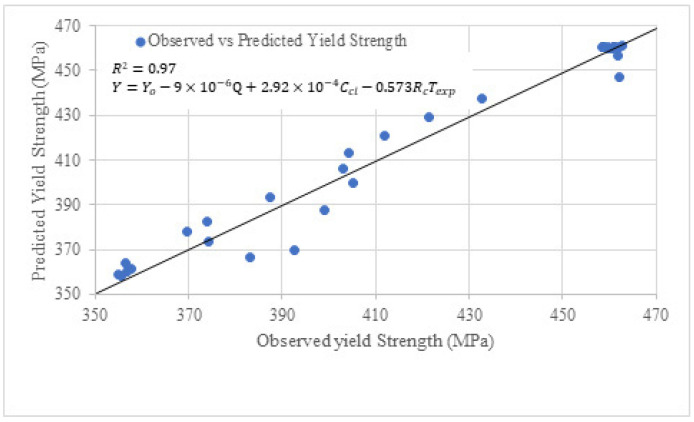
Yield strength model of bare steel bar under chloride exposure.

**Figure 20:**
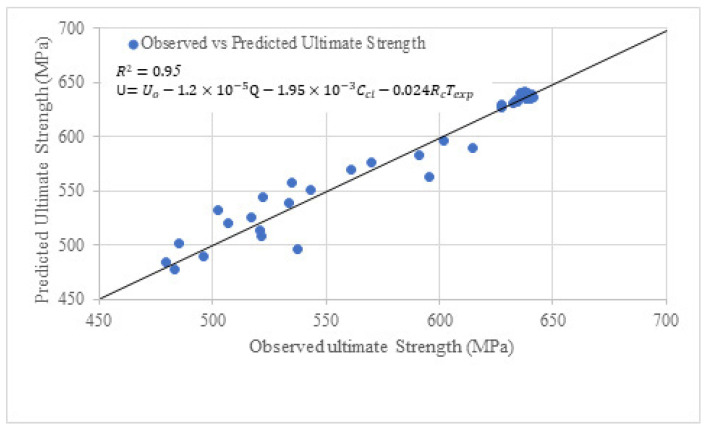
Ultimate strength model of bare steel bar under chloride exposure.

As it is evident that Q has the highest positive correlation with both strengths. Multiple linear regression analysis through least squares error has been used to determine the coefficients of independent variables. The significance level *p*-value 0.05 has been considered in this study, which means there is a 5% or less probability that the predicted values will deviate from the true value due to unseen factors. In other words, a maximum of five (05) predicted values out of (50) will not follow the model. The results of regression models in terms of the values of coefficients are shown in Figure 19 and Figure 20.

In Figure 19, the predicted and experimental yield strengths have been plotted. The value of *R*^2^ is 0.97, which shows that the equation is well fitted to the experimental value. All *p*-values are lower than or equal to 0.05; however, the *p*-value is relatively higher for the term $C_{cl}$. The higher *p*-value suggests that the term $C_{cl}$. It is not as significant as in the case of ultimate strength. The coefficient of the term $R_{c}T_{exp}$ suggests that the corrosion rate with exposure period has a major contribution in reducing the strength of bare steel bars, and it increases with exposure period.

In Figure 20, the predicted and experimental ultimate strength have been plotted. The value of R^2^ is 0.95, which shows that the equation is well fitted to the experimental value. All *p*-values are lower than or equal to 0.05; however, the *p*-value is relatively higher for the term $R_{c}T_{exp}$. The higher *p*-value suggests that the term ${\;R}_{c}T_{exp}$. It is not as significant as in the case of yield strength. The coefficient of the term $R_{c}T_{exp}$ suggests that the corrosion rate with exposure period has less contribution in reducing the ultimate tensile strength as compared to the yield strength.

## 7. Conclusions and Recommendations

The study highlights the synergistic deterioration mechanism of chloride in Karachi’s coastal environment. Under accelerated conditions, the average corrosion current density reached 1.35 µA/cm^2^ compared to 0.48 µA/cm^2^ in natural exposure. Tensile strength decreased by up to 1.6% after 9 months of natural exposure and 24.3% after 60 days of accelerated testing. Based on the experimental investigation of ASTM A615 grade 60 reinforcing steel bars exposed to natural and simulated chloride environments using Arabian seawater, the following conclusions are drawn:

1. Reinforcing bars exposed before concrete placement experience progressive corrosion that reduces cross-sectional area and alters mechanical performance, demonstrating that construction-stage exposure is not negligible in aggressive coastal climates.
2. Over 9 months of outdoor exposure, mass and diameter losses were small but consistent, corresponding to approximately 0.5% annual reduction in effective cross-sectional area. Associated reductions in yield and ultimate strength were limited and within experimental variability but followed a decreasing trend consistent with section loss.
3. Impressed current exposure in real seawater produced much higher corrosion rates, resulting in substantial diameter reduction (up to ~1 mm) and notable decreases in yield and ultimate tensile strength (up to about 20–25% within 60 days). This confirms the strong sensitivity of reinforcing steel to direct chloride environments in the absence of concrete protection.
4. Mechanical degradation trends correspond closely with measured diameter loss, indicating that a reduction in load-carrying area is the primary contributor to strength decrease. The possible microstructural or localised effects cannot be ruled out but were not quantified in this study.
5. SEM observations show porous, cracked, and loosely bonded corrosion products, suggesting that the rust layers do not form a stable protective barrier and allow continued corrosion progression.
6. A time equivalence relationship between simulated and natural exposure was derived based on mass and strength degradation trends. This equivalence is empirical and performance-oriented, not a claim of identical corrosion mechanisms or product composition.
7. Regression models relating exposure parameters to residual yield and ultimate strength showed strong agreement with experimental data, demonstrating potential for estimating mechanical performance loss due to pre-placement corrosion.
8. The findings highlight that reinforcing steel stored unprotected in marine environments may enter service with pre-existing damage. Construction management practices in coastal regions should therefore limit exposure duration or provide protective measures to reduce early corrosion.

Based on the findings of this study, the following recommendations are proposed:

1. Reinforcing steel intended for coastal construction should not be stored unprotected in open environments for extended periods, preferably not more than a month. Temporary protective measures or covered storage should be mandated in marine regions.
2. Design codes and construction guidelines should explicitly account for pre-embedding corrosion losses, particularly in aggressive coastal climates such as Karachi.
3. Accelerated corrosion testing protocols using real seawater should be adopted for durability assessment, as they realistically replicate marine exposure conditions.
4. Future research should:
  o Extend the study to bond behaviour between corroded bars and concrete.
  o Develop predictive models linking exposure duration, mass loss, and mechanical degradation.
  o Investigate the performance of coated or corrosion-resistant reinforcement under similar pre-exposure conditions.
5. Structural assessment and rehabilitation of coastal structures should consider initial steel degradation prior to concrete casting when estimating residual service life.

## Figures and Tables

**Table 1:** Environmental characteristics of Karachi (2024) [19], highlighting temperature, humidity, and rainfall conditions relevant to atmospheric corrosion severity.

Average Annual Temperature (°C)	Average Temperature of the Hottest Month (°C)	Average Temperature of the Coldest Month (°C)	Average Relative Humidity of the Hottest Month (%)	Average Relative Humidity of the Coldest Month (%)	Average Annual Rainfall (mm)	Average Annual Sunshine (h)
26.5	31	20	79	60	4.5	9

**Table 2:** Chloride concentration in groundwater and coastal environment of Karachi, indicating sustained exposure to aggressive chloride conditions [20,21,22].

Exposure Time	November 2024	December 2024	January 2025	February 2025	March 2025	April 2025	May 2025	June 2025	July 2025	August 2025
Cl Conc.	450	485	500	430	460	500	510	500	485	510

**Table 3:** Chemical composition of ASTM A615 grade 60 Reinforcing steel used in this study, confirming compliance with standard specification limits.

Carbon (C) (%)	Manganese (Mn) (%)	Sulphur (S) (%)	Phosphorous (%)	
			Test Results	ASTM A615
0.14	0.7	0.035	0.021	0.06

**Table 4:** Physico-chemical characteristics of Arabian seawater collected from Karachi coastline.

Characteristics	Values
Temperature (°C)	32.4
Colour value (Pt/Co).	48
Turbidity (NTU)	1.85
Conductivity (mg/L)	1207
pH	8.52
Total suspended solids (TSS)	843
Total dissolved solids (TDS)	55,873
Dissolved oxygen (mg/L)	5.35
Total nitrogen (mg/L)	0.576
Total phosphorous (mg/L)	0.264
Total chloride (mg/L)	20,000

**Table 5:** Evolution of tensile properties of reinforcing steel bars under natural chloride exposure.

Exposure Time	Yield Strength (MPa)	Ultimate Tensile Strength (MPa)	% Strain at Fracture	Change in Yield Strength (%)	Change in Ultimate Tensile Strength (%)
(Days)					
0	463	638	23.9	0	0
30	462	636	29.1	0.15	0.24
45	461	636	27.8	0.37	0.32
60	461	637	26.9	0.4	0.13
75	461	639	24.8	0.45	−0.12
90	461	638	24.8	0.46	−0.03
105	461	641	19.8	0.47	−0.48
120	460	638	20.2	0.51	0.03
135	460	637	19.9	0.54	0.16
150	460	641	12.7	0.59	−0.56
165	460	640	21.9	0.6	−0.38
180	460	638	18	0.62	−0.04
195	460	634	28	0.65	0.59
210	460	634	14.1	0.67	0.57
225	460	633	12.2	0.69	0.82
240	459	627	12.8	0.87	1.63
255	459	627	12.8	0.91	1.65
270	459	628	13.5	0.92	1.59

**Table 6:** Gravimetric mass loss of reinforcing steel bars subjected to natural chloride exposure.

ID No	Exposure (Days)	Bar Weight (g)		Weight Difference (g)	Diameter of Bars (mm)	Cross-Sectional Area (mm^2^)	Weight Loss (g/mm^2^)
		Before	After				
CL1	15	303	302	1.042	8.93	62.63	0.017
CL2	30	307	305	2.000	8.91	62.35	0.032
CL3	45	306	303	3.307	8.95	62.91	0.053
CL4	60	306	301	5.136	8.92	62.49	0.082
CL5	75	306	300	6.136	8.94	62.77	0.098
CL6	90	306	298	8.136	8.91	62.35	0.130
CL7	105	306	297	9.136	8.9	62.21	0.147
CL8	120	308	298	10.000	8.93	62.63	0.160
CL9	135	307	297	10.000	8.95	62.91	0.159
CL10	150	306	294	12.136	8.9	62.21	0.195
CL11	165	303	290	13.000	8.92	62.49	0.208
CL12	180	303	291	12.000	8.95	62.91	0.191
CL13	195	306	292	14.000	8.89	62.07	0.226
CL14	210	306	292	14.000	8.93	62.63	0.224
CL15	225	303	290	13.000	8.91	62.35	0.208
CL16	240	306	290	16.000	8.93	62.63	0.255
CL17	255	308	290	18.000	8.85	61.51	0.293
CL18	270	306	288	18.000	8.92	62.49	0.288

**Table 7:** Measured diameter reduction in reinforcing steel bars during natural chloride exposure.

Bare Bars (Natural Exposure)				
ID No	Exposure (Days)	Bar Diameter (mm)		Diameter Loss (mm)
		Before	After	
CL1	15	8.93	8.53	0.4
CL2	30	8.91	8.44	0.47
CL3	45	8.95	8.37	0.58
CL4	60	8.92	8.34	0.58
CL5	75	8.94	8.3	0.64
CL6	90	8.91	8.26	0.65
CL7	105	8.9	8.25	0.65
CL8	120	8.93	8.25	0.68
CL9	135	8.95	8.23	0.72
CL10	150	8.9	8.17	0.73
CL11	165	8.92	8.18	0.74
CL12	180	8.95	8.2	0.75
CL13	195	8.89	8.13	0.76
CL14	210	8.93	8.15	0.78
CL15	225	8.91	8.12	0.79
CL16	240	8.93	8.13	0.8
CL17	255	8.85	8.05	0.8
CL18	270	8.92	8.12	0.8

**Table 8:** Evolution of tensile properties of reinforcing steel bars under simulated chloride exposure.

Exposure Time (Days)	Yield Strength (N/mm^2^)	Ultimate Tensile Strength (N/mm^2^)	% Strain at Fracture	Reduction in Yield Strength (%)	Reduction in Ultimate Tensile Strength (%)
0	463	638	22.3	0	0
3	462	602	21.2	0.22	5.60
6	462	615	20.2	0.18	3.58
9	433	591	16.3	6.53	7.32
12	421	570	18.7	8.94	10.58
15	412	561	15.8	11.03	11.98
18	404	595	15.9	12.65	6.68
21	403	535	16.4	12.90	16.13
24	405	543	11.4	12.46	14.87
27	388	522	27.3	16.26	18.10
30	399	534	19.2	13.80	16.35
33	374	502	19.7	19.21	21.27
36	370	517	27.4	20.11	18.92
39	374	507	29.3	19.11	20.49
42	393	521	18.6	15.15	18.31
45	383	522	27.9	17.19	18.19
48	357	485	24.5	22.95	23.96
51	358	537	29.4	22.69	15.73
54	357	496	26.4	22.90	22.22
57	355	480	28.3	23.30	24.80
60	356	483	22.3	23.14	24.25

**Table 9:** Measured diameter reduction in reinforcing steel bars during simulated chloride exposure.

Bare Bars (Simulated Exposure)							
ID No	Exposure (Days)	Bar Weight (g)		Difference (gm)	Diameter of Bars	Cross-Sectional Area (mm^2^)	Weight Loss (g/mm^2^)
		Before	After		(mm)		
	0	260.21	260.21	0.00	8.76	60.27	0.00
CL1	3	261.40	261.03	0.37	8.75	60.13	0.01
CL2	6	257.40	257.16	0.24	8.75	60.16	0.00
CL3	9	259.20	242.43	16.77	8.47	56.34	0.30
CL4	12	261.30	238.26	23.04	8.36	54.88	0.42
CL5	15	263.20	234.71	28.49	8.26	53.62	0.53
CL6	18	258.70	226.00	32.70	8.19	52.65	0.62
CL7	21	260.50	227.14	33.36	8.18	52.49	0.64
CL8	24	261.40	229.20	32.20	8.20	52.76	0.61
CL9	27	263.60	221.51	42.09	8.02	50.47	0.83
CL10	30	264.50	228.81	35.69	8.13	51.95	0.69
CL11	33	257.80	208.02	49.78	7.87	48.69	1.02
CL12	36	258.40	206.29	52.11	7.83	48.15	1.08
CL13	39	259.20	209.69	49.51	7.88	48.75	1.02
CL14	42	261.70	222.50	39.20	8.07	51.14	0.77
CL15	45	262.30	217.78	44.52	7.97	49.91	0.89
CL16	48	257.80	198.29	59.51	7.69	46.44	1.28
CL17	51	263.20	204.36	58.84	7.70	46.59	1.26
CL18	54	259.20	199.82	59.38	7.69	46.47	1.28
CL19	57	261.60	201.18	60.42	7.67	46.22	1.31
CL20	60	262.70	202.71	59.99	7.68	46.32	1.30

**Table 10:** Measured diameter reduction in reinforcing steel bars in simulated chloride environment.

Bare Bars (Simulated Exposure)				
ID No	Exposure (Days)	Bar Diameter (mm)		Diameter Loss (mm)
		Before	After	
	0	8.76	8.76	0.00
CL1	3	8.76	8.75	0.01
CL2	6	8.76	8.75	0.01
CL3	9	8.76	8.47	0.29
CL4	12	8.76	8.36	0.40
CL5	15	8.76	8.26	0.50
CL6	18	8.76	8.19	0.57
CL7	21	8.76	8.18	0.58
CL8	24	8.76	8.20	0.56
CL9	27	8.76	8.02	0.74
CL10	30	8.76	8.13	0.63
CL11	33	8.76	7.87	0.89
CL12	36	8.76	7.83	0.93
CL13	39	8.76	7.88	0.88
CL14	42	8.76	8.07	0.69
CL15	45	8.76	7.97	0.79
CL16	48	8.76	7.69	1.07
CL17	51	8.76	7.70	1.06
CL18	54	8.76	7.69	1.07
CL19	57	8.76	7.67	1.09
CL20	60	8.76	7.68	1.08

**Table 11:** Correlation coefficients between exposure parameters and residual mechanical properties of reinforcing steel bars used for regression model development.

Variables for Yield Strength	$Y_{o}$	Q	$C_{cl}$	$R_{c}T_{exp}$
$Y_{o}$	1			
Q	0.782	1		
$C_{cl}$	−0.764	−0.891	1	
$R_{c}T_{exp}$	−0.973	−0.820	0.846	1
Variables for Yield Strength	$U_{o}$	Q	$C_{cl}$	$R_{c}T_{exp}$
$U_{o}$	1			
Q	0.782	1		
$C_{cl}$	0.441	0.523	1	
$R_{c}T_{exp}$	−0.954	−0.862	−0.428	1

## Data Availability

The original contributions presented in this study are included in the article. Further inquiries can be directed to the corresponding author.
